# Genetic Analysis of *PICK1* Gene in Alzheimer's Disease: A Study for Finding a New Gene Target

**DOI:** 10.3389/fneur.2018.01169

**Published:** 2019-01-09

**Authors:** Lingjia Xu, Yanxing Chen, Ting Shen, Caixiu Lin, Baorong Zhang

**Affiliations:** ^1^Department of Neurology, The Second Affiliated Hospital, School of Medicine, Zhejiang University, Hangzhou, China; ^2^Department of Neurology, The Second Hospital of Shaoxing, Shaoxing, China

**Keywords:** PICK1, Alzheimer's disease, single nucleotide polymorphism, gene target, genetic study

## Abstract

**Background:** Alzheimer's disease (AD) is a neurodegenerative disease with no effective treatment. Researchers have focused on exploring biomarkers for its early diagnosis, especially on finding a new gene target. Recent studies have shown that protein interacting with C-kinase-1(PICK1) is related to AD through regulating hippocampal synaptic plasticity. *PICK1* gene polymorphisms have been identified in psychological and other related disorders.

**Methods:** This study included 133 sporadic AD patients and 173 healthy controls. All coding exons and intron-exon boundaries of the *PICK1* gene were amplified by polymerase chain reaction (PCR), which were subsequently sequenced and analyzed.

**Results:** This is the first genetic association study to investigate the association between *PICK1* gene and AD risk in Chinese Han population. Seven single nucleotide polymorphisms (SNPs) were found in our research (rs397780637, rs713729, rs2076369, rs58230476, rs7289911, rs149474436; and rs146770324 for patient M1659 only). Frequencies of the T allele (*p* = 0.002; OR, 0.083; 95%CI, 0.011–0.634) and TT/TC genotypes (*p* = 0.001) of rs149474436 were lower in AD patients than in the controls. The GG homozygotes of rs397780637 were found to be associated with an increased risk of AD (*p* = 0.018) in *APOE*ε4 allele carriers, while the frequency of the T allele of rs149474436 was significantly lower among AD patients in *APOE*ε4 non-carriers (*p* = 0.005).

**Conclusions:** Our results suggest that *PICK1* gene SNPs are associated with AD susceptibility in East Asian population, T allele of rs149474436 may play as a protective factor while the rs397780637 GG homozygotes may be associated with an increased risk of AD. Further studies should be considered in a larger cohort of patients with diverse demographics.

## Introduction

Alzheimer's disease (AD) is a neurodegenerative disorder with irreversible memory decline and personality changes, which increases the burden of both the family and society. Pathogenic mechanisms of AD, especially its genetic susceptibilities are hot topics in this field. Recently, many rare variants have been identified as genetic markers of AD, such as *APP* (1), *TREM2* (2), *CD33*, and *MS4A* (3, 4). However, the results from different clinical centers are controversial. Apolipoprotein E (*APOE*) ε4 allele is so far, the strongest genetic risk factor for AD.

Protein interacting with C-kinase-1 (PICK1) is a peripheral membrane protein containing PSD95/DIgA/ZO-1 (PDZ) domain and Bin/amphiphysin/Rvs (BAR) domain (5). It is expressed in various tissues, particularly abundant in the brain and testis (6). In the central nervous system (CNS), PICK1 interacts with numerous neurotransmitter receptors, transporters, and enzymes, regulating their trafficking (7). Based on the interaction of PICK1 with CNS dopamine transporter system, the gene of *PICK1* has been implicated in schizophrenia and methamphetamine abusers, and positive results have been identified (8, 9). It has been shown that PICK1 also plays an important role in hippocampal synaptic plasticity, which makes it a potential target for the early diagnosis and treatment of AD. It regulates trafficking of α-amino-3-hydroxy-5-methyl-4-isoxazolepropionic acid receptor (AMPAR) and N-methyl-D-aspartate receptor (NMDAR) plasticity during hippocampal long-term depression (LTD) and long-term potentiation (LTP) (10). Another important mechanism underlying the role of PICK1 in cognition is about D-serine, a co-agonist of NMDAR, playing an important role in regulating synaptic plasticity of neuronal cells (11). Following the interaction between PICK1 and serine racemase, an enzyme converting L-serine to D-serine, protein kinase C (PKC) can be directed to its targets in cells. PKC can also regulate the activity of serine racemase and the levels of D-serine in the brain, indicating the regulation of PICK1 in NMDAR mediates neurotransmission and synaptic plasticity (12, 13).

Senile plaques, consisting of amyloid beta (Aβ), and neurofibrillary tangles, consisting of hyperphosphorylated tau, are the two main pathological hallmarks of AD (14). In Alfonso's studies, they found that the PDZ domain of PICK1 was required for Aβ in weakening synapses. Mice lacking PICK1 failed to depress synaptic transmission or reduce surface AMPAR subunit2 (15). Glycogen synthase kinase-3β (GSK-3β), a well-known serine/threonine kinase, phosphorylates various substrates including tau. It can also phosphorylate PICK1 during LTD induction. Furthermore, in tau knock-out mice, there was a loss of LTD, which inferred that PICK1 might regulate tau hyper-phosphorylation through GSK-3β during LTD in AD (15, 16). Therefore, it is of particular interest to investigate the association between the *PICK1* gene and the risk of AD.

The present study is a case-control study to investigate the association between *PICK1* gene polymorphisms and the risk of developing AD. We found that SNPs at the *PICK1* gene may modulate the risk of AD.

## Materials and Methods

### Case-Control Study

Subjects included in this case-control study were consisted of 133 sporadic AD patients (age 70.32 ± 9.63) and 173 controls (age 68.98 ± 9.42). The AD patients were enrolled from the Department of Neurology, Second Affiliated Hospital of Zhejiang University, School of Medicine. A clinical diagnosis of AD was established according to the criteria of the National Institute of Neurological and Communicative Disorders and Stroke/Alzheimer's Disease and Related Disorders Association (17, 18) by two experienced neurological physicians. Patients reported a family history and other neurological diseases were excluded. Healthy Individuals were recruited from the Health Examination Center in the same hospital and matched for gender, age, ethnicity, and area of residence. Informed consent was obtained from every participant. All work was conducted in accordance with the Declaration of Helsinki (1964). The study was approved by the Ethical Committee of the Second Affiliated Hospital, School of Medicine, Zhejiang University. Their demographic information is listed in Table 1.

**Table 1:** Clinical characteristics of AD patients and healthy controls.

	**AD**	**CON**	**Comparison**
Total samples	133	173
Male	72	94	*P* > 0.1
Female	61	79
Age (years, mean ± SD^a^)	70.32 ± 9.63	68.98 ± 9.42	T = 1.23 *p* = 0.22
MMSE^b^ score (mean ± SD)	15.40± 6.22	26.71 ± 1.89	*P* < 0.05

The table shows clinical characteristics of the 133 AD patients and173 healthy controls. There were no significant differences in age and sex between the two groups. AD patients showed a lower MMSE score compared to the controls.

a SD, standard deviation.

b *MMSE, Mini-Mental State Examination*.

### Genotype Analysis

Genomic DNA was isolated from 5 ml peripheral blood sample by standard procedure. The polymerase chain reaction (PCR) amplification of *APOE* gene was performed according to a previously described method (19). All coding exons and intron-exon boundaries of the *PICK1* gene were amplified by PCR. The primers used are listed in Table 2 (designed based on information about the *PICK1* gene obtained from a public database http://www.ncbi.nlm.nih.gov/; all primers were from Sangon Biotech (Shanghai) Co., Ltd.). The 25 μl reaction mixture included 1 μl forward primer, 1 μl reverse primer, 12.5 μl 2XHieff™ PCR Master Mix (10102ES03; YEASEN Biotech (Shanghai) Co., Ltd.), and 1 μl genomic DNA. The reaction conditions were as follows: (1) heated at 95°C for 5 min for denaturation; (2) subjected to 35 cycles of amplification by denaturation (95°C for 30 s); (3) primer annealing (annealing time for 30 s); (4) extension (72°C for 30–60 s); and (5) final extension (72°C for 10 min). Identification of the PCR-amplified products was followed by 2% agarose gel electrophoresis.

**Table 2:** PCR Primers of *PICK1* Gene.

**Region**	**Sense primers (5**′**-3**′**)**	**Anti-sense primers (5**′**-3**′**)**	**Product length (bp)**	**Tm^&^ (°C)**
Exon1-2	GCTCAGGGATGCTTTCGT	CCAGGGAGTCTTCCTCTATT	1,395	56
Exon3	CTGGGCAACAAGAGTAAGAC	CAGACAGAGGGCAAATAACA	595	56
Exon4	TGGGCAACAAGAGCGAAAC	CACAAGTCCTGGAGCACGATTA	605	52
Exon5	AGGAGTCTCAGTCCAGAACAGTCTTG	TTGGTCAGAGGTCAGAGCCCAC	321	52
Exon6	CTCCCTGTGCATGGAGGTAAGG	TGGTGACTTCTCAGTTCCACGG	317	56
Exon7	TGACCTCCCCTCTTCTTTGA	ATTTTGTAGGCTGGCATTCC	190	52
Exon8	CCCCATTCCGCATCACTCG	CCATCGCAAATCCCAGCACC	241	52
Exon9	GCCACCTCCACAAACCTTGACC	CCCCACCCTCACACGCCAGA	489	52
Exon10-11	TGACGCATCAGTGCCATTC	CACGGCTGTTCTTCATTCC	1,318	54
Exon12	CTTCACTCCTATGAGGCGCTT	CTCCCCGCTCCCAGTTCAGG	626	56
Exon13-1	CCCTGCCTCCGCCCCTTGCC	CTCCGTCCTCCCACGCACCCT	476	56
Exon13-2	GAGCCGTCCAGGGATACACGAG	CCTGCCACCTCCAAGTCCTTTC	390	56
Exon13-3	AGAGGGAGAGCTTGGTCTCTGGACC	AAGGAGGGTCTGAAGCCACTGCGAC	358	56

*The table shows the PCR primers used in the study to search for SNPs in all exons and boundaries between exons and introns of PICK1 Gene*.

& *Melting temperature*.

Each PCR-amplified product was directly sequenced on an ABI3730xlDNAAnalyzer automated sequencer (Applied Biosystems, Foster City, CA). DNAStar was used for sequence alignment and analysis (DNAStar, Inc., Madison, WI) by two analysts independently. The consequences of variants at protein sequence were predicted according to the *PICK1* cDNA sequences in GenBank (accession numbers NM_012407 and NP_036539.1, respectively).

## Statistical Analysis

All statistical analyses were performed using SPSS version 20.0. SNPStats (available online at http://bioinfo.iconcologia.net/SNPstats) was used to analyze the Hardy–Weinberg equilibrium (HWE) for each SNP in both AD patients and controls. Differences in allele and genotype frequencies between the AD patients and controls were assessed using chi-squared test and fisher's exact test. Linkage disequilibrium and haplotype analysis were analyzed by SHEsis (an online analysis tool: http://analysis.bio-x.cn/myAnalysis.php) (20, 21). In all tests, a *p* < 0.05 was considered statistically significant. The odds ratio (OR; 95% confidence interval) of AD cases to controls was calculated.

## Results

**Figure 1:**
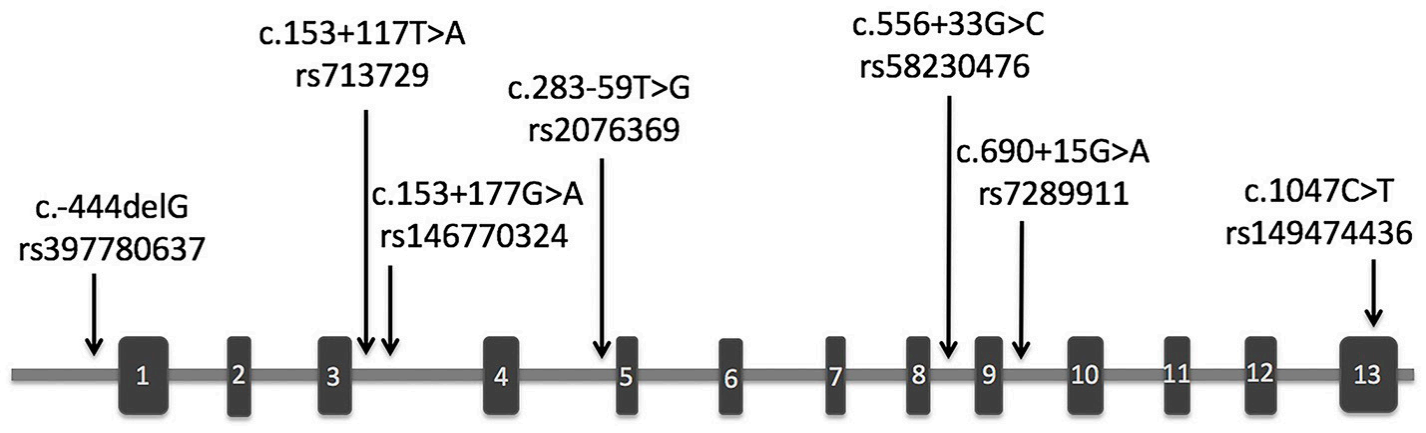
Schematic representation of *PICK1* gene with location of the SNPs found in our study. The rectangles and horizontal lines respectively represent exons and introns of *PICK1* gene. Black arrows indicate the location of SNPs detected in this gene in our study.

The demographic and clinical characteristics of AD cases and controls are shown in Table 1. There were no significant differences in age and sex between the two groups. The distribution of the selected SNPs was in HWE in both groups (*p* > 0.05). Table 3 shows the genotypic and allelic associations of *PICK1* with *APOE* gene in AD patients and controls. As anticipated, the frequency of the *APOE*ε4 allele was significantly higher in AD patients than in controls. No pathogenic mutation in *PICK1* gene but 7 SNPs were found in our research (rs397780637, rs713729, rs2076369, rs58230476, rs7289911, rs149474436; and rs146770324 for patient M1659 only), indicating that *PICK1* gene mutation may be rare in AD patients (Figure 1). With regard to rs149474436, we observed a significantly lower frequency of the T allele (*p* = 0.002; OR, 0.083; 95%CI, 0.011–0.634) and TT/TC genotypes (*p* = 0.001) in AD patients than in the controls. When the data were stratified by *APOE*ε4 status in Table 4, the GG homozygotes of rs397780637 were found to be associated with an increased risk of AD (*p* = 0.018) in *APOE*ε4 allele carriers. The frequency of T allele of the SNP rs149474436 was significantly lower among the AD patients than the controls in *APOE*ε4 non-carriers (*p* = 0.005). With regard to other SNPs, there were no significant differences in either allelic or genotypic frequency between the two groups.

**Table 3:** Genotypic and allelic frequencies of *APOE* and *PICK1* gene variants in AD patients and controls.

	***n***	**Genotypes,** ***n* (%)**			***p***	**Alleles,** ***n*(%)**		***p***	**OR (95%CI)**
*APOE*		ε4, ε4	ε4,–	–,–	< 0.001	ε4+	ε4-	< 0.001	2.785 (1.786–4.342)
AD	133	10 (7.5)	45 (33.8)	78 (58.6)		65 (24.4)	201 (75.6)	
CON	173	1 (0.6)	34 (19.7)	138 (79.8)		36 (10.4)	310 (89.6)	
rs397780637		–,–	–,G	GG	0.439	–	G	0.195	0.800 (0.571–1.121)
AD	133	15 (11.3)	55 (41.4)	63 (47.4)		85 (32.0)	181 (68.0)	
CON	173	25 (14.5)	78 (45.1)	70 (40.5)		128 (37.0)	218 (63.0)	
rs713729		AA	AT	TT	0.549	A	T	0.273	1.243 (0.842–1.833)
AD	133	8 (6.0)	46 (34.6)	79 (59.4)		62 (23.3)	204 (76.7)	
CON	173	7 (4.0)	54 (31.2)	112 (64.7)		68 (19.7)	278 (80.3)	
rs146770324^#^		AA	AG	GG	NA	A	G	0.435	NA
AD	133	0 (0)	1 (0.8)	132 (99.2)		1 (0.4)	265 (99.6)	
CON	173	0 (0)	0 (0)	173 (100)		0 (0)	346 (100.0)	
rs2076369		GG	GT	TT	0.977	G	T	0.908	1.020 (0.727–1.432)
AD	133	56 (42.1)	66 (49.6)	11 (8.3)		178 (66.9)	88 (33.2)	
CON	173	71 (41.0)	88 (50.9)	14 (8.1)		230 (66.5)	116 (33.5)	
rs58230476		CC	CG	GG	0.232	C	G	0.886	1.031 (0.681–1.559)
AD	133	86 (64.7)	46 (34.6)	1 (0.8)		218 (82.0)	48 (18.0)	
CON	173	115 (66.5)	52 (30.1)	6 (3.5)		282 (81.5)	64 (18.5)	
rs7289911		AA	AG	GG	0.329	A	G	0.146	1.506 (0.864–2.624)
AD	133	2 (1.5)	25 (18.8)	106 (79.7)		29 (10.9)	237 (89.1)	
CON	173	1 (0.6)	24 (13.9)	148 (85.5)		26 (7.5)	320 (92.5)	
rs149474436		TT	TC	CC	0.001	T	C	0.002	0.083 (0.011–0.634)
AD	133	0 (0)	1 (0.8)	132 (99.2)		1 (0.4)	265 (99.6)	
CON	173	0 (0)	15 (8.7)	158 (91.3)		15 (4.3)	331 (95.7)	

*There list the genotypic and allelic frequencies of the APOE and PICK1 gene variants in AD patients and controls. 7 SNPs (rs397780637, rs713729, rs146770324, rs2076369, rs58230476, rs7289911, and rs149474436) were found, the third to the sixth columns contain information of the genotypic data and the seventh to the tenth columns describe the allelic data. Results showed frequencies of the T allele (p = 0.002; OR, 0.083; 95%CI, 0.011–0.634) and TT/TC genotypes (p = 0.001) of rs149474436 were lower in the AD patients than in the controls. OR, odds ratio; CI, confidence interval; #rs146770324 was found in patient M1659 only; NA, not available*.

**Table 4:** Distribution of the SNPs in AD patients and controls after stratification by *APOE*ε4 status.

	***N***	**Genotypes** ***n*** **(%)**			***p***	**Alleles** ***n*** **(%)**		***p***	**OR (95% CI)**
rs397780637		–,–	–,G	GG		–	G	
*APOE*ε4(–)	216				0.324			0.826	0.955 (0.635–1.437)
AD	78	8 (10.3)	40 (51.3)	30 (38.5)		56 (35.9)	100 (64.1)	
CON	138	22 (15.9)	58 (42.0)	58 (42.0)		102 (37.0)	174 (63.0)	
*APOE*ε4(+)	90				0.018			0.126	0.606 (0.318–1.154)
AD	55	7 (12.7)	15 (27.3)	33 (60.0)		29 (26.4)	81 (73.6)	
CON	35	3 (8.6)	20 (57.1)	12 (34.3)		26 (37.1)	44 (62.9)	
rs713729		AA	AT	TT		A	T	
*APOE*ε4(–)	216				0.485			0.242	1.324 (0.826–2.121)
AD	78	5 (6.4)	28 (35.9)	45 (57.7)		38 (24.4)	118 (75.6)	
CON	138	5 (3.6)	44 (31.9)	89 (64.5)		54 (19.6)	222 (80.4)	
*APOE*ε4(+)	90				0.934			0.771	1.116 (0.533–2.340)
AD	55	3 (5.5)	18 (32.7)	34 (61.8)		24 (21.8)	86 (78.2)	
CON	35	2 (5.7)	10 (28.6)	23 (65.7)		14 (20.0)	56 (80.0)	
rs146770324		AA	AG	GG		A	G	
*APOE*ε4(–)	216				NA			NA	–
AD	78	0 (0)	0 (0)	78 (100.0)		0 (0)	156 (100.0)	
CON	138	0 (0)	0 (0)	138 (100.0)		0 (0)	276 (100.0)	
*APOE*ε4(+)	90				NA			NA	–
AD	55	0 (0)	1 (1.8)	54 (98.2)		1 (0.9)	109 (99.1)	
CON	35	0 (0)	0 (0)	35 (100.0)		0 (0)	70 (100.0)	
rs2076369		GG	GT	TT		G	T	
*APOE*ε4(–)	216				0.997			0.947	1.015 (0.662–1.556)
AD	78	36 (46.2)	37 (47.4)	5 (6.4)		109 (69.9)	47 (30.1)	
CON	138	63 (45.7)	66 (47.8)	9 (6.5)		192 (69.6)	84 (30.4)	
*APOE*ε4(+)	90				0.398			0.261	1.417 (0.771–2.605)
AD	55	20 (36.4)	29 (52.7)	6 (10.9)		69 (62.7)	41 (37.3)	
CON	35	8 (22.9)	22 (62.9)	5 (14.3)		38 (54.3)	32 (45.7)	
rs58230476		CC	CG	GG		C	G	
*APOE*ε4(–)	216				0.373			0.848	1.053 (0.624–1.777)
AD	78	52 (66.7)	26 (33.3)	0 (0)		130 (83.3)	26 (16.7)	
CON	138	94 (68.1)	40 (29.0)	4 (2.9)		228 (82.6)	48 (17.4)	
*APOE*ε4(+)	90				0.718			0.647	1.185 (0.572–2.454)
AD	55	34 (61.8)	20 (36.4)	1 (1.8)		88 (80.0)	22 (20.0)	
CON	35	21 (60.0)	12 (34.3)	2 (5.7)		54 (77.1)	16 (22.9)	
rs7289911		AA	AG	GG		A	G	
*APOE*ε4(–)	216				0.182			0.091	1.711 (0.914–3.204)
AD	78	1 (1.3)	19 (24.4)	58 (74.4)		21 (13.5)	135 (86.5)	
CON	138	1 (0.7)	21 (15.2)	116 (84.1)		23 (8.3)	253 (91.7)	
*APOE*ε4(+)	90				1.000			0.533	1.752 (0.449–6.840)
AD	55	1 (1.8)	6 (10.9)	48 (87.3)		8 (7.3)	102 (92.7)	
CON	35	0 (0)	3 (8.6)	32 (91.4)		3 (4.3)	67 (95.7)	
rs149474436		TT	TC	CC		T	C	
*APOE*ε4(–)	216				0.005			0.005	NA
AD	78	0 (0)	0 (0)	78 (100.0)		0 (0)	156 (100.0)	
CON	138	0 (0)	12 (8.7)	126 (91.3)		12 (4.3)	264 (95.7)	
*APOE*ε4(+)	90				0.295			0.301	0.205 (0.021–2.010)
AD	55	0 (0)	1 (1.8)	54 (98.2)		1 (0.9)	109 (90.1)	
CON	35	0 (0)	3 (8.6)	32 (91.4)		3 (4.3)	67 (95.7)	

*After stratification by APOEε4 status, genotypic, and allelic data of the PICK1 gene SNPs are listed below. The GG homozygotes of rs397780637 were found to be associated with an increased risk of AD (p = 0.018) in APOEε4 allele carriers, while the frequency of the T allele of rs149474436 was significantly lower among the AD patients in APOEε4 non-carriers (p = 0.005). OR, odds ratio; CI, confidence interval; NA, not available*.

Sex differences in risk for AD related to *PICK1* gene polymorphisms rs397780637 and rs149474436 are listed in Table 5. The male patients showed a higher G allele of rs397780637 (*p* = 0.048; OR, 0.634; 95%CI, 0.402–0.998), a higher C allele (*p* = 0.020) and CC homozygotes (*p* = 0.019) of rs149474436 compared to the male healthy controls. It is an interesting finding that the male with above SNPs seem to have a higher risk for AD.

**Table 5:** Frequencies of genotypes and alleles of SNP rs397780637 and rs149474436 after stratification of data by sex.

	***N***	**Genotypes** ***n*** **(%)**			***p***	**Alleles** ***n*** **(%)**		***p***	**OR (95% CI)**
rs397780637		–,–	–,G	GG		–	G	
Male	166				0.109			0.048	0.634 (0.402–0.998)
AD	72	11 (15.3)	24 (33.3)	37 (51.4)		46 (31.9)	98 (68.1)	
CON	94	19 (20.2)	42 (44.7)	33 (35.1)		80 (42.6)	108 (57.4)	
Female	140				0.825			0.776	1.077 (0.647–1.793)
AD	61	4 (6.6)	31 (50.8)	26 (42.6)		39 (32.0)	83 (68.0)	
CON	79	6 (7.6)	36 (45.6)	37 (46.8)		48 (30.4)	110 (69.6)	
rs149474436		TT	TC	CC		T	C	
Male	166				0.019			0.020	NA
AD	72	0 (0)	0 (0)	72 (100.00)		0 (0)	144 (100.00)	
CON	94	0 (0)	7 (7.4)	87 (92.6)		7 (3.7)	181 (96.3)	
Female	140				0.077			0.082	0.155 (0.019–1.256)
AD	61	0 (0)	1 (1.6)	60 (98.4)		1 (0.8)	121 (99.2)	
CON	79	0 (0)	8 (10.1)	71 (89.9)		8 (5.1)	150 (94.9)	

*Sex differences in risk for AD related to PICK1 gene polymorphisms rs397780637 and rs149474436 are listed below. Results revealed the male patients had a higher G allele of rs397780637 (p = 0.048; OR, 0.634; 95%CI, 0.402–0.998), a higher C allele (p = 0.020), and CC homozygotes (p = 0.019) of rs149474436 compared to the male healthy controls. OR, odds ratio; CI, confidence interval*.

**Figure 2:**
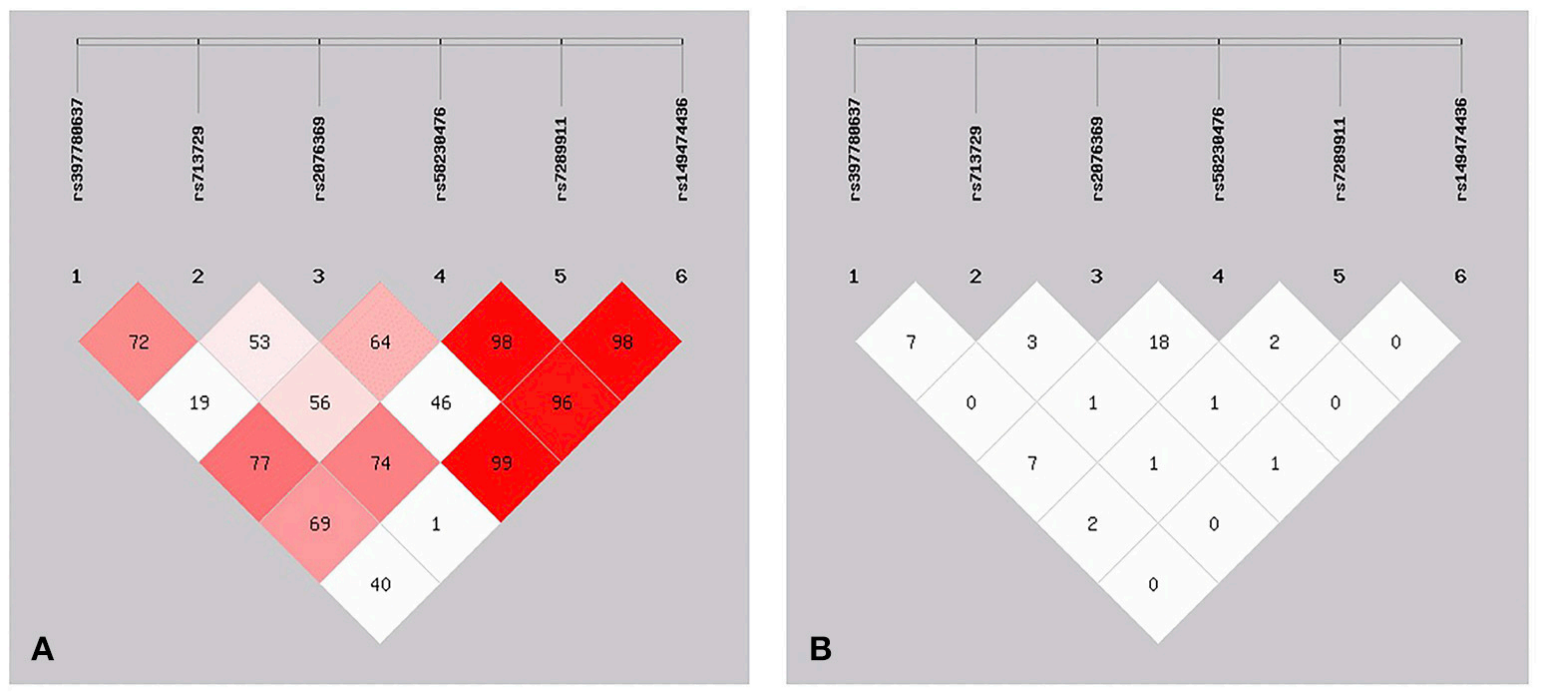
The patterns of linkage disequilibrium of the six SNPs in the *PICK1* gene, with their D′ **(A)** and *r*^2^ **(B)** values.

Of these SNPs, SNP146770324 was only found in patient M1659. The frequency was too low, so we chose the other six SNPs for better analysis of the linkage disequilibrium. As shown in Figure 2, three of these six SNPs showed low frequencies of recombination using parameters D′ (Figure 2A). However, *r*^2^ analysis revealed negative results (Figure 2B). These conflicting results might be related with the relatively small sample size and low frequencies of the SNPs. Further studies with larger sample size and various demographic groups are urgently needed to further validate the linkage disequilibrium.

## Discussion

We found seven SNPs in the present study. T allele of rs149474436 may play as a protective factor while the rs397780637 GG homozygotes are found to be associated with an increased risk of AD. AD is a neurodegenerative disorder, the prevalence of which increases with aging. It has recently been shown that PICK1 plays an important role in AMPAR mediated synaptic plasticity during normal aging. The decrease in PICK1 level, which was accompanied by a reduction in GluR2, might lead to their altered subcellular distribution, and subsequently, deficits in synaptic plasticity in the hippocampus of aged rats (22). Research from Volk et al. showed that genetic deletion of *PICK1* at hippocampal synapses in adult and juvenile mice might selectively influence their learning and memory through regulating AMPAR trafficking. Loss of PICK1 impaired some forms of LTP and LTD, and also the inhibitory avoidance learning only in adult mice (10). It is known that AMPAR dysfunction is related with many neurodegenerative diseases, including AD (23). PICK1 has been shown to interact with D-serine and serine racemase in the brain (12). A recent study showed that combined application of D-serine related drugs and classical antipsychotic drugs could improve the cognition of patients with schizophrenia (24), indicating the potential role of PICK1 in cognitive function. Brain insulin resistance and impaired glucose tolerance is now suggested to be one of the molecular pathogenesis of AD (25). Holst et al. found that *PICK1* mRNA was up-regulated in type 2 diabetes and in high-fat-diet-induced obese mice, indicating the role of PICK1 in regulating insulin sensitivity (26). Besides, PICK1-GluR2 interaction has also been reported in the regulation of ischemia induced AMPAR trafficking, leading to delayed neuronal death following insults (27). AD is multi-factorial and is now accepted to be caused by multiple causes. Given the role of PICK1 in the regulation of the above-mentioned pathophysiologic processes, we assume PICK1 may also be dynamically involved in the AD pathogenesis. Further studies are needed to confirm this.

Therefore, it is possible that AD-associated symptoms can be partially associated with PICK1 dysfunction, caused by its genetic variations. The *PICK1* gene has been mapped to chromosome 22q13.1 (10), and is identified to play a role in conferring susceptibility to schizophrenia (8, 28). In a recent study, Chen et al. (29) explored the role of polymorphisms of *PICK1* gene (rs2076369, rs3952) in cognitive functions in schizophrenic patients. They enrolled 302 patients and analyzed the differences of cognitive functions and clinical symptoms among different genetic groups. They found that patients with rs2076369 GT heterozygotes showed better performance than TT homozygotes, suggesting the association between *PICK1* gene SNPs and cognitive decline in schizophrenic patients. Therefore, we proposed that *PICK1* genetic polymorphisms may be also associated with AD risk. In this study, we used direct sequencing which can provide stronger power to detect association than single-variant analyses, especially when these variants are rare or novel.

The present study shows the differences in genetic backgrounds between AD patients and healthy controls. However, the physiological and pathogenic roles of the *PICK1* SNPs examined in this study (rs397780637: c.-444delG in intron variant, upstream variant 2KB, and rs149474436:c.1047C>T in the downstream variant defined as non-coding transcript variant, synonymous codon) are unknown. It is possible that it may affect the pre-mRNA splicing of the *PICK1* intron, affecting its splicing or protein translation through regulating the secondary structure of mRNA. It may also influence AD risk via its linkage disequilibrium with other functional variants and nearby genes. The receptor and transporter interactions mainly occur at the PDZ and BAR domains of PICK1. Therefore, further studies aimed at identifying the mutation in these two domains seem highly warranted. However, this study has some limitations. Firstly, no association between the *PICK1* polymorphisms and disease severity (such as MMSE score) is identified. Therefore, it seems less likely that the *PICK1* gene is involved in the deterioration of the disease. But further studies are needed to confirm this finding. Secondly, in case-control association studies, risk of spurious associations that results from chance findings or stratification effects in the sample collection may also exist. In the present study, the sample size was relatively small and type I error cannot be ruled out, thus subgroup studies are hard to perform. Finally, participants included in this study were mainly from east China. Studies with larger sample size carried out in various demographic groups are needed to further validate the association between the variants evaluated in this study and AD risk.

## Conclusion

In conclusion, the present case-control study suggests that *PICK1* gene may be a new gene target for AD. This finding can provide a basis for future genetic studies on AD and other neurological disorders. Studies with larger sample size, various demographic groups, and whole gene sequencing technique are needed to confirm the association between *PICK1* gene and AD risk. Mechanistic studies are also needed to elucidate its role in AD pathogenesis.

## Availability of Data and Material

All data arising from this study is contained within the manuscript. The genotyping data generated during this study can be accessed through contacting the corresponding authors.

## Author Contributions

LX and YC designed this study. TS and CL gathered the demographic information. LX did the genotypic analysis and interpreted the data and drafted the manuscript. YC and BZ approved the final version to be published.

### Conflict of Interest Statement

The authors declare that the research was conducted in the absence of any commercial or financial relationships that could be construed as a potential conflict of interest.

## Abbreviations

AD: Alzheimer's disease
PICK1: protein interacting with C-kinase-1
AMPAR: α-amino-3-hydroxy-5-methyl-4-isoxazolepropionic acid receptor
SNP: single nucleotide polymorphism
APOE: apolipoprotein E
PDZ: PSD95/DIgA/ZO-1
BAR: Bin/amphiphysin/Rvs
CNS: central nervous system
NMDAR: N-methyl-D-aspartate receptor
LTD: long-term depression
LTP: long-term potentiation
Aβ: Amyloid-beta protein
GSK-3β: glycogen synthase kinase-3β
PCR: polymerase chain reaction
HWE: Hardy–Weinberg equilibrium.
